# Time to peak bilirubin concentration and advanced AKI were associated with increased mortality in rheumatic heart valve replacement surgery patients with severe postoperative hyperbilirubinemia: a retrospective cohort study

**DOI:** 10.1186/s12872-020-01830-5

**Published:** 2021-01-06

**Authors:** Xiaolan Chen, Ming Bai, Lijuan Zhao, Yan Yu, Yuan Yue, Shiren Sun, Xiangmei Chen

**Affiliations:** 1 The Nephrology Department of Xijing Hospital, The Fourth Military Medical University, No. 127 Changle West, Road, Xi’an, 710032 China; 2 State Key Laboratory of Kidney Disease, Department of Nephrology, Chinese People’s Liberation Army General Hospital and Military Medical Postgraduate College, 28th Fuxing Road, Beijing, 100853 China

**Keywords:** Heart valve surgery, Rheumatic heart disease, Acute kidney injury, Continuous renal replacement therapy, Hyperbilirubinemia

## Abstract

**Background:**

Hyperbilirubinemia after heart valve surgery (HVS) with cardiopulmonary bypass is frequently observed and associated with worse outcomes. We investigated the characteristics and prognosis of patients with severe hyperbilirubinemia after HVS for rheumatic heart disease (RHD) to identify the clinical outcomes and potential risk factors.

**Methods:**

Between 2015 and 2018, patients who underwent HVS in the cardiac surgery intensive care unit of our hospital were retrospectively screened. Risk factors for acute kidney injury (AKI), the requirement for continuous renal replacement therapy (CRRT), and in-hospital and long-term mortality were identified by univariate and multivariate analyses. The patient survival proportion was graphically presented with the Kaplan–Meier method.

**Results:**

A total of 149 patients who underwent HVS for RHD and had severe postoperative hyperbilirubinemia were included. Of the included patients, 80.5% developed postoperative AKI, and 18.1% required CRRT. The in-hospital mortality was 30.2%. Backward logistic regression analysis showed that the time to peak TB concentration (odds ratio [OR] 1.557, 95% confidence interval [CI] 1.259–1.926; *P* < 0.001) and advanced AKI (stage 2 and 3 AKI) (OR 19.408, 95% CI 6.553–57.482; *P* < 0.001) were independent predictors for in-hospital mortality. The cutoff value of the time to peak TB levels for predicting in-hospital mortality was 5 postoperative days.

**Conclusions:**

Severe postoperative hyperbilirubinemia is a life-threatening complication in patients who undergo HVS for RHD. Patients whose bilirubin levels continued to increase past the 5th postoperative day and who had advanced AKI (stages 2 and 3) were associated with a higher risk of mortality.

## Background

The global prevalence of rheumatic heart disease (RHD) was estimated to be 39,345,369 cases by 2017 [1]. RHD is primarily caused by acute rheumatic fever in developing countries and results in permanent damage to the heart valve. Heart valve surgery (HVS) under cardiopulmonary bypass (CPB) is an effective treatment for RHD. However, despite the latest developments in surgical techniques and perioperative management, HVS for RHD still cause a high burden of morbidity and mortality [2, 3].

The incidence of hyperbilirubinemia after HVS has been reported to be approximately 30–40% [4–9] and is higher than that of some other types of heart surgery, including coronary artery bypass grafting and surgery for congenital heart disease [4, 10–12]. Previous studies indicated that transient mild hyperbilirubinemia was usually associated with a favorable prognosis, while late-occurring severe hyperbilirubinemia (5 times the normal upper limit) was associated with high mortality and morbidity for patients with cardiac surgery [10]. This is mainly because mild hyperbilirubinemia is usually reversible and temporary, which may be caused by hemolysis, hepatic hypoperfusion, gaseous microemboli, and the necessity for blood transfusions during CPB. However, severe hyperbilirubinemia could be associated with oxidative stress and cell apoptosis[13], which mainly cause respiratory failure and neurological dysfunction and consequently develop multiple organ failure (MOF) and increase the risk of short-term mortality [10, 14]. Additionally, our previous study showed that the prognosis of patients with severe postoperative hyperbilirubinemia remains significantly different. Some patients recovered within a few weeks, while others progressed to MOF, which led to an increase in short-term mortality [15]. To explore of the characteristics, prognosis, and risk factors for in-hospital and long-term mortality is helpful for clinicians understand the prognosis for patients with severe postoperative hyperbilirubinemia, which is useful for patient consultation as well as decision making. However, to date, there are relatively few reports on the characteristics and outcomes of patients with severe postoperative hyperbilirubinemia after HVS for RHD.

Hence, the current study was designed to explore the characteristics and to inquire the predictors for in-hospital and long-term mortality in RHD surgery patients with severe postoperative hyperbilirubinemia.

## Methods

### Study patients

In the retrospective cohort study, consecutive patients who underwent HVS, had severe hyperbilirubinemia, and admitted to the cardiac surgery intensive care unit (ICU) of our hospital between January 2015 and December 2018 were screened. RHD was diagnosed according to previous acute rheumatic fever and/or symptoms of precordial abnormalities and the presence of heart murmur, more importantly, according to echocardiographic findings [16]. Severe hyperbilirubinemia was defined as a TB concentration greater than 85.5 µmol/l during the hospital stay. The following conditions are excluded: (1) age < 16 years old; (2) HVS surgery for non-RHD; (3) severe hyperbilirubinemia before surgery; and (4) history of dialysis before HVS surgery. The cohort study was approved by the institutional research ethics committee of Xijing Hospital and had a waiver of individual informed consent due to the retrospective study design.

### Data collection

Demographic and operative data previously reported to be associated with postoperative hyperbilirubinemia and AKI after HVS were collected via retrospective chart review from our hospital’s electronic medical record system. Routine laboratory data before surgery (that closest to the time of surgery) and the postoperative period were recorded. Urine output on every postoperative day and blood pressure on the first day after the surgery were also recorded. Moreover, consistent with our previous research, the acute physiology and chronic health evaluation II (APACHE II) score, European system for cardiac operative risk evaluation II (EuroSCORE II), model for end-stage liver disease (MELD) score, and sequential organ failure assessment (SOFA) score were calculated to assess the severity of the disease [15].

### Postoperative outcomes and data definition

Postoperative outcomes, including the use of vasoactive agents, hospital stays, ICU monitoring time, the duration of ventilatory support, the amount of blood transfusion, AKI incidence, and the requirement of CRRT, bilirubin adsorption (BA), and plasma exchange (PE), were recorded. In our clinical practice, the living patients were routinely followed up at 1, 3, and 6 months and then every 6 months after the surgery.

The definition and classification of AKI and chronic kidney disease (CKD) are based on the Kidney Disease Improving Global Outcomes (KDIGO) criteria [17]. The Chronic Kidney Disease Epidemiology Collaboration equation (CKD-EPI) was performed to calculate the glomerular filtration rate (GFR). The latest SCr concentration before surgery was regarded as the preoperative SCr concentration. The major indications including progressive AKI, fluid overload, severe metabolic acidosis, and hyperkalemia were considered for the initiation of CRRT [17].

### Statistical analysis

All quantitative data are described as the means ± standard deviation (SD), and qualitative data are depicted as numbers (n) and percentages (%). Quantitative data was compared by Student’s t-test and qualitative data was compared by the χ^2^ test or Fisher exact test. Factors related to study endpoints in univariate analysis and important clinical parameters were put into backward logistic regression analysis or Cox regression analysis to seek the independent predictors. Relation analysis and collinearity diagnosis were also performed, and only one of the variables with a significant correlation was included in the multivariate regression analysis. Similar to our previous studies, the Kaplan–Meier method was utilized to evaluate cumulative survival and the log-rank test was used to evaluate the differences between the two groups in the cumulative survival[15]. To further evaluate the effect of time to peak TB concentration on predicting patient in-hospital mortality, the area under curve of the receiver operating characteristic (AUC-ROC) was computed and the Youden index was employed to assess the optimal cutoff values of time to peak TB concentration. Bilateral *P* < 0.05 was regarded as statistically significant for all analyses. Statistical analysis was performed by using the IBM SPSS version 22.0 software package (SPSS Chicago, IL).

## Result

### Patient characteristics

**Fig. 1:**
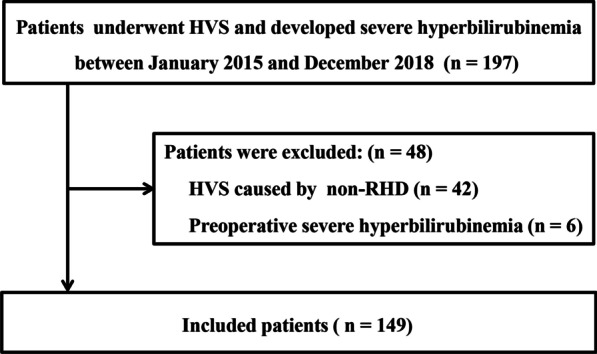
Patient inclusion flow chart

After the screening, 197 patients were considered candidates for inclusion. Of these patients, 42 and 6 patients were excluded due to HVS for non-RHD and preoperative TB ≥ 85.5 µmol/l, respectively. Finally, 149 patients were included in our current study (Fig. 1).

Table 1 presented the baseline characteristics of all patients. The overall mean age was 52.1 ± 9.3 years, and 35.6% were male. Forty-five (30.2%) patients had eGFR < 60 ml/min before HVS surgery. About half of the included patients had aortic valve, mitral valve, and tricuspid valve replacement at the same time. The mean preoperative TB concentration was 28.9 ± 15.9 µmol/l, the occurrence time of severe hyperbilirubinemia was 2.3 ± 1.6 days, and the average time to peak TB concentration was 4.2 ± 3.4 days after HVS. The median follow-up time after HVS was 21.7 (0.1–57.2) months. A total of two patients were lost to follow-up, and the time to loss of follow-up was 0.42 and 21.2 months after HVS for RHD, respectively.

**Table 1:** Baseline characteristics in patients developing hyperbilirubinemia

Variables	Value
*Preoperative*	
Age (years)	52.11 ± 9.28
Male, n (%)	53 (35.6%)
Hypertension, n (%)	11 (7.4%)
Diabetes, n (%)	2 (1.3%)
Atrial fibrillation, n (%)	53 (35.6%)
eGFR (ml/min)	67.05 ± 15.06
CKD (eGFR < 60 ml/min)	45 (30.2%)
Stage 3 (30 ml/min ≤ eGFR < 60 ml/min)	43 (28.9%)
Stage 4 (15 ml/min ≤ eGFR < 30 ml/min)	1 (0.7%)
Stage 5 (eGFR < 15 ml/min	1 (0.7%)
EuroSCORE II	1.8 ± 1.3
APACHE II score	6.29 ± 2.78
MELD score	9.88 ± 4.47
SOFA score	1.87 ± 1.54
MAP (mmHg)	86.95 ± 12.14
TB (µmol/l)	28.86 ± 15.93
WBC (10^9^/l)	6.20 ± 2.21
Hb (g/l)	136.30 ± 19.33
PLT (10^9^ /l)	168.87 ± 58.26
Cr (µmol/l)	98.80 ± 38.05
Cys-C (mg/l)	1.169 ± 0.537
PT (s)	13.49 ± 4.36
*Intra-operative*	
Type of surgery	
Single aortic valve, n (%)	9 (6.0%)
Single mitral valve, n (%)	25 (16.8%)
Single tricuspid valve, n (%)	3 (2.0%)
Aortic valve + mitral valve, n (%)	19 (12.8%)
Aortic valve + tricuspid valve, n (%)	1 (0.7%)
Mitral valve + tricuspid valve, n (%)	28 (18.8%)
Aortic valve + mitral valve + tricuspid valve, n (%)	63 (42.3%)
Valve + CABG, n (%)	3 (2.0%)
Operation duration (h)	4.32 ± 1.48
CPB time (min)	161.16 ± 60.46
ACC time (min)	89.40 ± 35.04
Blood transfusion requirement (U)	4.86 ± 7.08
Number of valve replacements	2.14 ± 0.83
*Postoperative*	
APACHE II score	17.77 ± 1.92
SOFA score	11.38 ± 2.44
MELD score	16.48 ± 4.49
TB (µmol /l)	69.80 ± 28.11
CB (µmol /l)	29.72 ± 13.02
WBC (10^9^/l)	14.48 ± 5.35
Hb (g/l)	119.41 ± 19.07
PLT (10^9^/l)	124.39 ± 52.73
Cr (µmol/l)	137.07 ± 35.24
PT (s)	13.60 ± 2.43
Peak TB level (µmol/l)	155.34 ± 82.11
Peak direct bilirubin level (µmol/l)	98.10 ± 65.90
Peak indirect bilirubin level (µmol/l)	57.24 ± 29.00
Time to peak TB (d)	4.18 ± 3.42

ACC, aortic cross clamp; APACHEII, acute physiology and chronic health evaluation II; CABG, coronary artery bypass grafting; CPB, cardiopulmonary bypass; Cys-c, cystatin C; eGFR, estimated glomerular filtration rate; Hb, hemoglobin; MAP, mean arterial pressure; MELD, model for end-stage liver disease; min, minute; PLT, platelet; PT, prothrombin time; SCr, serum creatinine; SOFA, sequential organ failure assessment; TB, total bilirubin; WBC, white blood cell

### Postoperative AKI

AKI after HVS was observed in 120 patients (80.5%), 69 patients (46.3%) were categorized as AKI stage 1, 20 patients (13.4%) as AKI stage 2, and 31 patients (20.8%) as AKI stage 3 (Table 2). Univariate analysis showed that age, preoperative hemoglobin (Hb), preoperative cystatin C (Cys-C), operation duration, CPB time, aortic cross-clamp (ACC) time, number of valve replacements, peak TB concentration, and time to peak TB concentration were significantly associated with AKI after HVS. Multivariable logistic regression analysis showed that preoperative Hb (OR 0.962, 95% CI 0.936–0.989; *P* = 0.006), age (OR 1.058, 95% CI 1.002–1.119; *P* = 0.044), CPB time (OR 1.017, 95% CI 1.005–1.029; *P* = 0.005), and the number of valve replacements (OR 2.024, 95% CI 1.144–3.582; *P* = 0.015) were independent predictors of postoperative AKI (Additional file 1: Table 1).

**Table 2:** Outcomes of the included patients

Variable	Value
In-hospital mortality, n (%)	45 (30.2%)
Cause of death	
Multiple organ failure, n (%)	25 (55.6%)
Heart failure, n (%)	16 (35.6%)
Hemorrhagic shock, n (%)	2 (4.4%)
Sepsis, n (%)	2 (4.4%)
In hospital time (d)	16.20 ± 4.90
Onset time of hyperbilirubinemia (d)	2.32 ± 1.57
ICU stay time (d)	5.09 ± 5.47
Postoperative AKI, n (%)	120 (80.5%)
Stage of AKI	
Stage 1, n (%)	69 (46.3%)
Stage 2, n (%)	20 (13.4%)
Stage 3, n (%)	31 (20.8%)
CRRT, n (%)	27 (18.1%)
Use of ECMO, n (%)	9 (6.0%)
Use of IABP, n (%)	4 (2.7%)
Use of tracheotomy, n (%)	5 (3.4%)
Use of vasoactive agent, n (%)	109 (73.2%)
Duration of mechanical ventilation (d)	2.83 ± 3.14
The amount of blood transfusion (U)	31.32 ± 33.60

AKI, acute kidney injury; BA, bilirubin adsorption; CRRT, continuous renal replacement therapy; ECMO, extracorporeal membrane oxygenation; IABP, intra-aortic balloon pump; ICU, intensive care unit; PE, plasma exchange

### Postoperative CRRT

In the current study, 22 (18.1%) patients received CRRT. Univariate logistic analysis revealed that the preoperative APACHE II score, platelet count, Hb concentration, left ventricular ejection fraction (LVEF), and Cys-C were significantly associated with postoperative CRRT. Multivariate logistic analysis revealed that preoperative Cys-C concentration (OR 29.530, 95% CI 3.998–218.125; *P* = 0.001) was the only independent predictor of postoperative CRRT (Additional file 1: Table 1).

### In-hospital and 30-day mortality

**Fig. 2:**
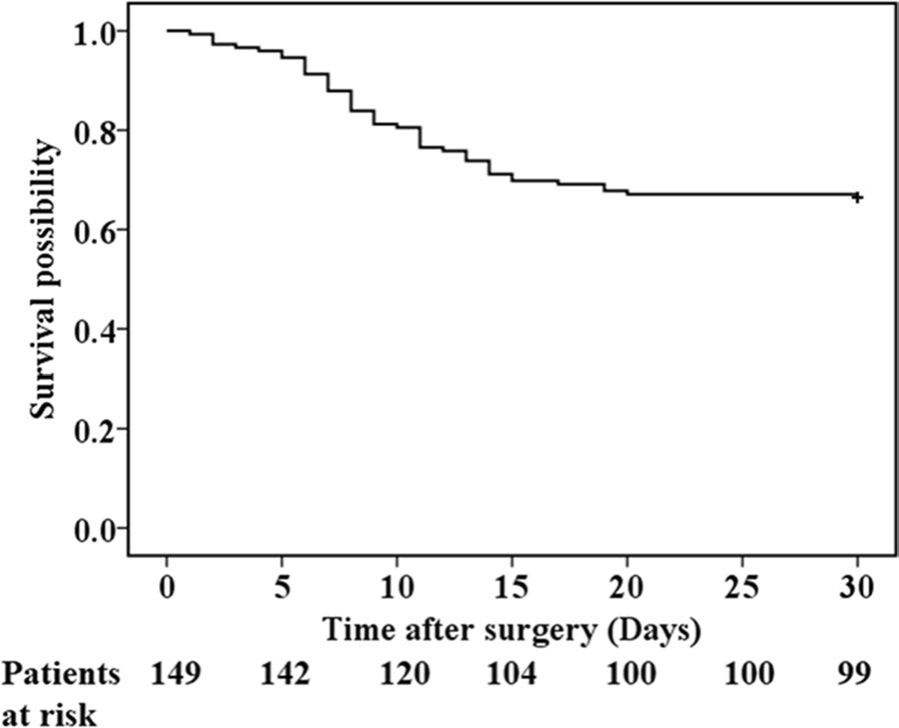
Kaplan–Meier curves for 30-day mortality

The overall in-hospital and 30-day mortality rates were 30.2% and 33.5%, respectively. The Kaplan–Meier survival curve of 30-day mortality was presented Fig. 2. The main causes of mortality were MOF (55.6%) and heart failure (35.6%). Other causes of mortality included hemorrhagic shock (4.4%) and sepsis (4.4%). Univariate logistic analysis found that 20 factors were related to in-hospital mortality. Multivariate logistic analysis found that the time to peak TB concentration (OR 1.557, 95% CI 1.259–1.926; *P* < 0.001) and advanced AKI (stage 2 and 3 AKI, OR 19.408, 95% CI 6.553–57.482; *P* < 0.001, Table 3) were independent risk factors for in-hospital mortality.

**Table 3:** Logistic regression analysis for in-hospital mortality

Characteristic	Univariate logistic regression		Multivariate logistic regression (model 1)		Multivariate logistic regression (model 2)	
	OR (95%CI)	*P* value	OR (95%CI)	*P* value	OR (95%CI)	*P* value
Age	1.55 (1.011–1.101)	0.014				
Preoperative						
APACHE II score	1.194 (1.051–1.356)	0.006				
CKD (eGFR < 60 ml/min)	1.233 (0.582–2.614)	0.584				
EuroSCORE II	1.435 (1.103–1.867)	0.007				
Intraoperative						
Operative time	1.570 (1.208 – 2.041)	0.001				
CPB time	1.007 (1.001–1.013)	0.018				
Amount of blood transfusion	1.078 (1.021 – 1.139)	0.007			1.078 (1.000–1.162)	0.049
Postoperative						
MAP	0.962 (0.927–0.998)	0.038				
The total amount of blood transfusion	1.077 (1.050–1.103)	< 0.001				
Mechanical ventilation time	2.734 (1.903–3.928)	< 0.001				
SOFA score	1.538 (1.283–1.845)	< 0.001				
Hb	0.976 (0.957–0.995)	0.015				
PLT	0.985 (0.977–0.993)	< 0.001				
AKI	7.539 (1.709–33.251)	0.008				
Advanced AKI	29.732 (11.507–76.821)	< 0.001	19.408 (6.553–57.482)	< 0.001	16.560 (5.154–53.212)	< 0.001
Use of CRRT	18.939 (6.485 – 55.313)	0.001				
Use of PE/BA	15.846 (1.848 – 135.879)	0.012				
Use of ECMO	22.270 (2.693–184.155)	0.004				
Peak TB concentration	1.018 (1.001–1.025)	< 0.001				
Peak direct bilirubin concentration	1.024 (1.015–1.033)	< 0.001				
Peak indirect bilirubin concentration	1.013 (1.001–1.026)	0.035				
Time to peak TB concentration	1.808 (1.458–2.242)	< 0.001	1.557 (1.259–1.926)	< 0.001		
Time to peak TB concentration ≥ 5 days	25.850 (10.218–65.399)	< 0.001			22.384 (6.767–74.035)	< 0.001
ICU stay time	1.285 (1.152–1.433)	< 0.001				

ACC, aortic cross clamp; AKI, acute kidney injury; APACHEII, acute physiology and chronic health evaluation II; BA, bilirubin adsorption; CPB, cardiopulmonary bypass; CRRT, continuous renal replacement therapy; ECMO, extracorporeal membrane oxygenation; eGFR, estimated glomerular filtration rate; Hb, hemoglobin; ICU, intensive care unit; MAP, mean arterial pressure; PE, plasma exchange; PLT, platelet; SOFA, sequential organ failure assessment; TB, total bilirubin

ROC curve analysis showed that the time to peak TB concentration after HVS (AUC = 0.835, 95% CI 0.753–0.916). The sensitivity and specificity was 73.3% and 90.4%, respectively (Additional file 2: Fig. 1). Additionally, we found patients with a time to peak TB concentration ≥ 5 days had a relatively higher risk of in-hospital mortality (OR 22.384, 95% CI 6.767–74.035; *P* < 0.001, Table 3, model 2) than patients with a time to peak TB concentration < 5 days in multivariate logistic regression analysis.

### Long-term mortality

**Fig. 3:**
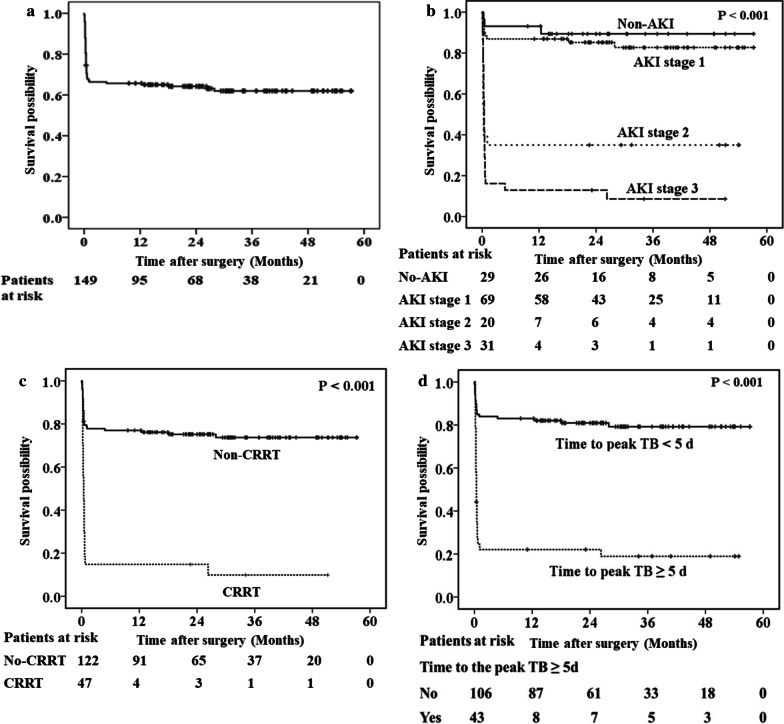
Long-term survival results of (**a**) all patients; **b** patients without AKI and those with stage 1, 2, or 3 AKI; **c** patients without the use of CRRT and those with CRRT; **d** patients with a time to peak TB concentration ≥ 5 d and those with a time to peak TB concentration < 5 d

Ten patients died during follow-up. Figure 3a showed that the cumulative mortality rate at 1 year, 2 year, and 3 year were 34.3%, 35.8%, and 38.0%, respectively. Univariate Cox regression analysis indicated 21 risk factors for long-term mortality, including postoperative AKI, requirement of CRRT, and time to peak TB concentration ≥ 5 days (Fig. 3b-d). Multivariate Cox regression analysis revealed that advanced AKI (stage 2 and 3 AKI, HR 7.379, 95% CI 3.791–14.364, *P* < 0.001), the amount of blood transfusion during surgery (HR 1.051, 95% CI 1.012–1.091; *P* = 0.01), and the mechanical ventilation time (HR 1.078, 95% CI 1.017–1.143; *P* = 0.011) were independent risk factors for long-term mortality in patients with severe hyperbilirubinemia after HVS for RHD (Table 4).

**Table 4:** Cox regression analysis for long-term mortality

Variables	Univariate Cox regression		Multivariate Cox regression	
	HR (95%CI)	*P* value	HR (95%CI)	*P* value
*Preoperative*				
Age	1.014 (1.011–1.073)	0.008		
APACHE II score	1.122 (1.060–1.188)	< 0.001		
CKD (eGFR < 60 ml/min)	0.914 (0.511–1.636)	0.763		
EuroSCORE II	1.325 (1.110–1.583)	0.002		
*Intraoperative*				
Operation duration	1.248 (1.098–1.414)	0.001		
CPB time	1.005 (1.002–1.008)	0.001		
The amount of blood transfusion	1.704 (1.037–1.112)	< 0.001	1.051 (1.012–1.091)	0.01
*Postoperative*				
SOFA score	1.356 (1.202–1.531)	< 0.001		
MELD score	1.086 (1.009–1.169)	0.028		
Hb	0.981 (0.968–0.995)	0.008		
PLT	0.991 (0.986–0.997)	0.002		
Cr	1.009 (1.002–1.016)	0.008		
Peak TB level	1.005 (1.003–1.008)	< 0.001		
Time to peak TB level	1.142 (1.089–1.198)	< 0.001		
AKI	5.19 (1.620–16.628)	0.006		
Advanced AKI	10.374 (5.606–19.199)	< 0.001	7.379 (3.791–14.364)	< 0.001
CRRT	5.64 (3.259–9.763)	< 0.001		
Use of ECMO	4.641 (2.240–9.612)	< 0.001		
PE/BA	2.791 (1.186–6.597)	0.019		
Onset time of hyperbilirubinemia	1.218 (1.119–1.326)	< 0.001		
Duration of mechanical ventilation	1.186 (1.128–1.246)	< 0.001	1.078 (1.017–1.143)	0.011
The total amount of blood transfusion	1.019 (1.014–1.024)	< 0.001		
ICU stay time	1.044 (1.019–1.070)	0.001		

AKI, acute kidney injury; APACHEII, acute physiology and chronic health evaluation II; BA, bilirubin adsorption; CPB, cardiopulmonary bypass; Cr, creatinine; CRRT, continuous renal replacement therapy; ECMO, extracorporeal membrane oxygenation; eGFR, estimated glomerular filtration rate; Hb, hemoglobin; ICU, intensive care unit; MAP, mean arterial pressure; PE, plasma exchange; PLT, platelet; SOFA, sequential organ failure assessment; TB, total bilirubin

## Discussion

It has been previously proved that the use of CPB is related to early hyperbilirubinemia and transient liver dysfunction [14, 18]. Nonetheless, the mortality rates of patients with severe hyperbilirubinemia were heterogeneous in previous reports. At present, there are limited data about the characteristics and risk factors for patients with severe hyperbilirubinemia after HVS for RHD. In this study, several important findings were as follows: First, the development of AKI and the use of CRRT in our cohort with severe hyperbilirubinemia were relatively higher than those in previous studies of HVS patients with and without hyperbilirubinemia, and the in-hospital mortality of our cohort was prominently higher than that in previous studies of patients who underwent HVS with all levels of hyperbilirubinemia. Second, age, preoperative Hb concentration, CPB time, and number of valve replacements were independent predictors for postoperative AKI, and an independent predictor for postoperative CRRT was the preoperative Cys-C concentration. Third, a time to peak TB concentration ≥ 5 days and the occurrence of postoperative advanced AKI (stages 2 and 3) were significantly associated with increased mortality.

### Patients with RHD with severe postoperative hyperbilirubinemia were associated with worse prognosis

The results of our current cohort of RHD patients with severe postoperative hyperbilirubinemia indicated an occurrence of postoperative AKI of 80.5%, a need for CRRT of 18.1%, and an in-hospital mortality of 30.2%. However, in previous studies of patients who underwent HVS with and without postoperative hyperbilirubinemia, the incidence of AKI ranged from 6.1% to 76% [19, 20], and the requirement of CRRT was approximately 3%. Additionally, the reported mortality in patients with all levels of postoperative hyperbilirubinemia after HVS was 15.9% [21]. The difference was most likely because all of the included patients with RHD in our current study had severe hyperbilirubinemia. The severe hyperbilirubinemia after HVS for RHD might be caused by the severity of the RHD itself and HVS injuries. The causes of poor prognosis have been described in our previous study [15]. In short, hyperbilirubinemia has been shown to promote apoptosis and aggravate renal ischemia–reperfusion injury in animal models [22]. Furthermore, previous studies further revealed that the incidence of hyperbilirubinemia was associated with postoperative AKI in cardiac surgery patients as well [23]. Severe Hyperbilirubinemia might cause cell apoptosis of the brain and aggravate the inflammatory response [24], which might be one of the potential causes of the poor prognosis in our current study with severe postoperative hyperbilirubinemia.

### Risk factors for postoperative AKI and CRRT

Our present study found that age and preoperative Hb were independent risk factors for AKI, which was consistent with previous studies in cardiac surgery patients [25–27]. Che et al*.* [28] showed that the incidence risk of postoperative AKI increased by 1.35 times per 10-year increase in the age of patients with CPB surgery [28]. Older patients commonly have worse basic structural and functional changes in the kidney, which may further aggravate the development of AKI after HVS for RHD. Patients with low preoperative Hb concentrations had a high risk of postoperative AKI, which might be related to the reduction in renal oxygen delivery [29].

Preoperative eGFR < 60 ml/min was not associated with AKI or CRRT. In clinical practice, we observed that poor preoperative kidney function (eGFR < 60 ml/min) could be reversed after the relief of RHD symptoms by HVS in some patients. Most likely, the occurrence of AKI was mainly related to injury severity during HVS instead of baseline kidney function. A systematic review [30] of cardiac surgery patients showed that the use of CPB was associated with the development of intravascular hemolysis, which could cause tubular epithelial cell injury and lead to renal insufficiency [31]. Moreover, it has been reported that surgical injury to tissues and the exposure of blood to the CPB pump and circuit could activate multiple inflammatory pathways and increase proinflammatory cytokines concentration [32]. This was further confirmed in our findings, where the correlation between CPB time and AKI was confirmed by multivariate regression analysis. Furthermore, the increased number of valve replacements was identified as an independent risk factor for AKI. Multiple valve replacement surgery is often associated with a longer CPB time and longer renal ischemia time, which could be a potential mechanism for the increased injury of the kidney. Therefore, surgeons could improve the prognosis of patients with RHD by improving their surgical methods and reducing the CPB time.

Cys-C was identified as an independent predictor for advanced AKI requiring CRRT. Cys-C was reported to be more precise in estimating the GFR and has a higher correlation with the gold standard method of GFR estimation, including DTPA scan and iohexol‐based clearance [33, 34]. The Cys-C concentration is mainly determined by glomerular filtration and is regarded as an early marker of glomerular filtration dysfunction [33]. Additionally, Belcher et al.[35] found that the change in Cys-C concentration occurred much earlier in AKI than the change in SCr concentration and was closely associated with the eventual requirement of CRRT or progression of AKI.

### Risk factors for in-hospital and long-term mortality

For patients with hyperbilirubinemia after CPB surgery, Farag et al*.*[10] indicated that the time to peak TB concentration was significantly relevant to in-hospital mortality. In our current study, we found the time to peak TB concentration was an independent predictor for in-hospital mortality for severe hyperbilirubinemia patients after HVS for RHD as well. Additionally, we found that the optimal cutoff value of the time to peak TB concentration for predicting in-hospital mortality was 5 days after surgery. Survival analysis demonstrated that patients with peak TB 5 or more days from surgery had significantly increased long-term mortality compared with those with peak TB within 5 days of surgery. Different time courses for postoperative severe hyperbilirubinemia suggested that the underlying mechanisms might not be the same. Mastoraki et al.[36] found that most of the patients with early mild hyperbilirubinemia could recover spontaneously when cardiac output was sufficient and oxygen delivery was adequate. Therefore, they considered that early mild postoperative hyperbilirubinemia might be caused by hemolysis, hypothermia, and hypotension during CPB surgery [37]. However, late severe postoperative hyperbilirubinemia was most likely related to liver dysfunction and resulted in poor prognosis, and the occurrence of liver dysfunction might be caused by persistent cardiac failure or sepsis[9, 15]. Therefore, monitoring heart function and maintaining hemodynamic stability after HVS for RHD are of great concern to prevent further deterioration.

Furthermore, a high concentration of bilirubin is a cytotoxic substance that can result in mitochondrial dysfunction and organ dysfunction. Currently, bilirubin clearance systems, including prometheus therapy, molecular adsorbent recirculation systems, plasma exchange, and bilirubin adsorption, have been proven to be effective in reducing serum bilirubin levels and improving patient survival for patients with liver failure [38–42]. However, the timing of the initiation of these methods for patients with RHD with severe postoperative hyperbilirubinemia is still uncertain and needs further evaluation.

Consistent with previous studies of patients who underwent cardiac surgery [43, 44], advanced AKI (stage 2 and stage 3) was also identified as an independent risk factor for in-hospital and long-term mortality in our present study. Our present study also indicated that an increased amount of blood transfusion during operation and prolonged length of mechanical ventilation were associated with increased long-term mortality as well. The increased amount of blood transfusion during operation and the prolonged length of mechanical ventilation usually indicate unstable circulatory and respiratory functions, respectively. These parameters represented the severity of the patients and were reasonably related to patient mortality [45, 46].

### Study limitations

There are some limitations to the current study. First, the retrospective design was one of the main limitations of our current study. The results might be affected by unknown confounders. Furthermore, the SCr concentration on admission was considered the baseline renal function. Some patients had elevated SCr at baseline, which would lead to the underestimation of the number of patients with AKI. Finally, the limited sample size might be associated with increased system errors. Further prospective multicenter studies with larger samples are required to gain stronger evidence.

## Conclusions

The development of severe hyperbilirubinemia is a prevalent threat in patients with RHD who undergo HVS. A lower Hb concentration, older age, longer CPB time, and an increased number of valve replacements were independent risk factors for AKI in patients with RHD with severe hyperbilirubinemia. Patients whose bilirubin levels continued to increase past the 5th postoperative day and who had advanced AKI (stages 2 and 3) were associated with a high risk of mortality.

## Supplementary information

**Additional file 1.** **Table 1:** Logistic regression analysis for postoperative AKI and CRRT.

**Additional file 2.** **Figure 1:** Receiver operator curve (ROC) analysis of the time to peak TBconcentration predicting in-hospital mortality; AUC, area under the curve; CI, confidence interval.

## Data Availability

The datasets used and/or analysed during the current study are available from the corresponding author on reasonable request.

## Abbreviations

AKI: Acute kidney injury
ACC: Aortic cross clamp
APACHE II: Acute physiology and chronic health evaluation II
BA: Bilirubin adsorption
CPB: Cardiopulmonary bypass
CRRT: Continuous renal replacement therapy
Cys-C: Cystatin C
eGFR: Estimated glomerular filtration rate
EuroSCORE II: European system for cardiac operative risk evaluation II
HVS: Heart valve surgery
ICU: Intensive care unit
MAP: Mean arterial pressure
MELD: Model for end-stage liver disease
MODS: Multiple organ dysfunction syndromes
PE: Plasma exchange
RHD: Rheumatic heart disease
SCr: Serum creatinine
SOFA: Sequential organ failure assessment
TB: Total bilirubin
