# Supplementary material for: Time to peak bilirubin concentration and advanced AKI were associated with increased mortality in rheumatic heart valve replacement surgery patients with severe postoperative hyperbilirubinemia: a retrospective cohort study
Source: BMC Cardiovasc Disord. 2021 Jan 6;21:16. doi: 10.1186/s12872-020-01830-5 (PMC7789141; doi:10.1186/s12872-020-01830-5)
Supplement: Supplementary file 1 — Additional file 1. Table 1: Logistic regression analysis for postoperative AKI and CRRT. [file 12872_2020_1830_MOESM1_ESM.docx]

| **Supplementary Table 1.** Logistic regression analysis for postoperative AKI and CRRT | | | | |
| --- | --- | --- | --- | --- |
| **Characteristic** | **Univariate logistic regression** | | **Multivariate logistic regression** | |
|  | **OR (95%CI）** | ***P* value** | **OR (95%CI）** | ***P* value** |
| **AKI** |  |  |  |  |
| Age | 1.071 (1.023 - 1.112) | 0.003 | 1.058 (1.002 - 1.119) | 0.044 |
| CKD (eGFR < 60 mL/min) | 1.846 (0.695 - 4.900) | 0.219 |  |  |
| Preoperative Hb | 0.970 (0.947 - 0.995) | 0.017 | 0.962 (0.936 - 0.989) | 0.006 |
| Preoperative Cys-C | 28.699 (3.006 - 274.018) | 0.012 |  |  |
| Number of valve replacements | 1.036 (1.006 - 1.067) | 0.019 | 2.024 (1.144 - 3.582) | 0.015 |
| Operation time | 2.679 (1.664 - 4.312) | < 0.001 |  |  |
| CPB time | 1.018 (1.007 - 1.029) | 0.001 | 1.017 (1.005 - 1.029) | 0.005 |
| ACC time | 1.024 (1.009 - 1.039) | 0.002 |  |  |
| Peak TB concentration | 1.009 (1.000 - 1.017) | 0.049 |  |  |
| Time to peak TB concentration | 1.425 (1.087 - 1.867) | 0.010 |  |  |
| **CRRT** |  |  |  |  |
| Preoperative LVEF (%) | 0.926 (0.871 - 0.985) | 0.015 |  |  |
| Preoperative APACHE Ⅱ | 1.198 (1.041 - 1.377) | 0.012 |  |  |
| Preoperative Hb | 0.996 (0.945 - 0.988) | 0.002 |  |  |
| Preoperative PLT | 0.990 (0.981 - 0.998) | 0.021 |  |  |
| Preoperative Cys-C | 16.679 (3.035 - 91.652) | 0.001 | 29.530 (3.998-218.125) | 0.001 |
| ACC, aortic cross clamp; APACHE Ⅱ, acute physiology and chronic health evaluation II; eGFR, estimated glomerular filtration rate; CI, confidence interval; CPB, cardiopulmonary bypass; Cys-C, cystatin C; Hb, hemoglobin; LVEF, left ventricular ejection fraction; OR, odds ratio; PLT, platelet; TB, total bilirubin | | | | |
