# Supplementary figures and images for: Time to peak bilirubin concentration and advanced AKI were associated with increased mortality in rheumatic heart valve replacement surgery patients with severe postoperative hyperbilirubinemia: a retrospective cohort study
Source: BMC Cardiovasc Disord. 2021 Jan 6;21:16. doi: 10.1186/s12872-020-01830-5 (PMC7789141; doi:10.1186/s12872-020-01830-5)

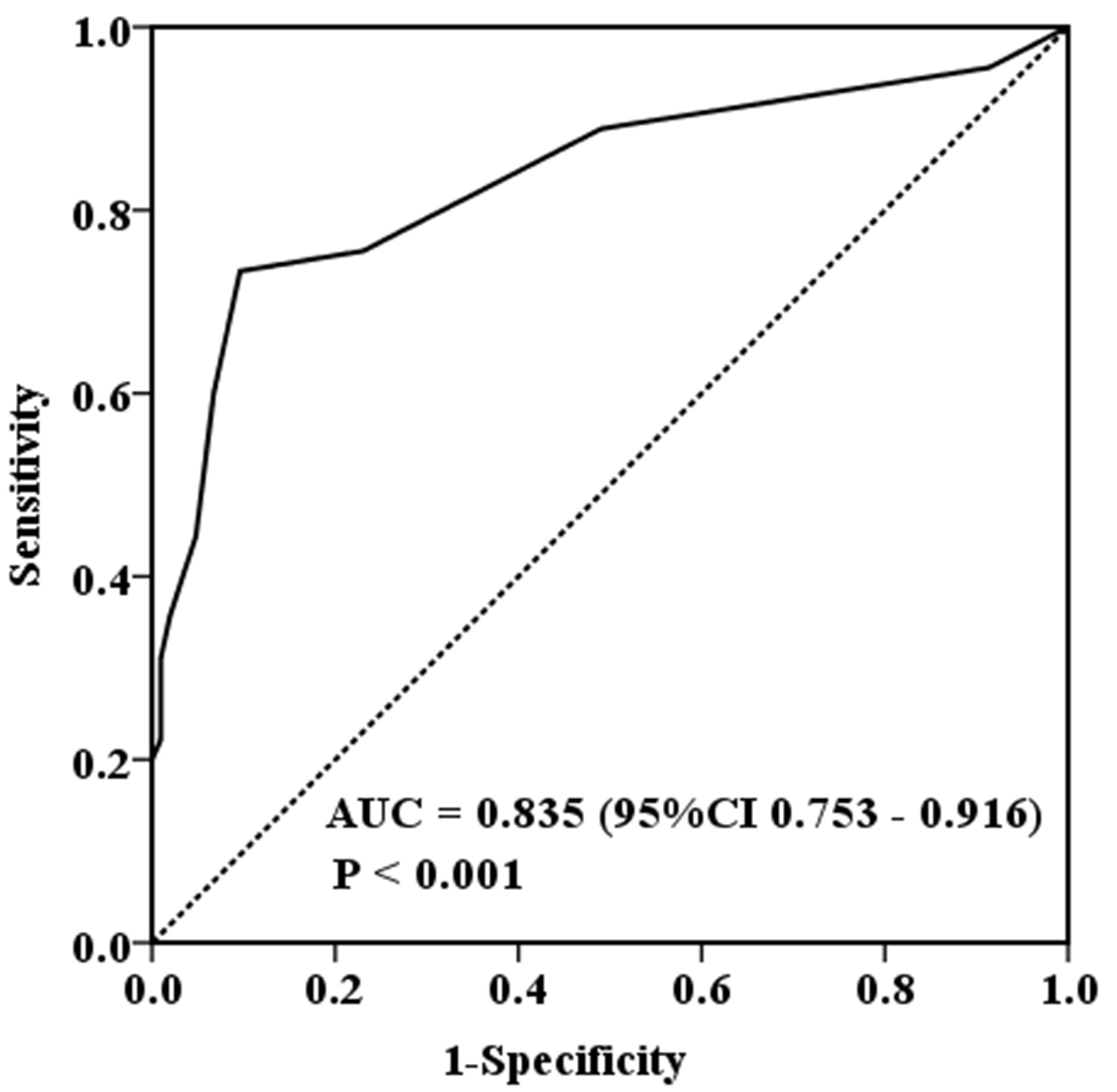

Supplement: Supplementary file 2 — Additional file 2. Figure 1: Receiver operator curve (ROC) analysis of the time to peak TBconcentration predicting in-hospital mortality; AUC, area under the curve; CI, confidence interval. [file 12872_2020_1830_MOESM2_ESM.tif]
